# PEEK and Hyaluronan-Based 3D Printed Structures: Promising Combination to Improve Bone Regeneration

**DOI:** 10.3390/molecules27248749

**Published:** 2022-12-09

**Authors:** Letizia Ferroni, Ugo D’Amora, Sara Leo, Elena Tremoli, Maria Grazia Raucci, Alfredo Ronca, Luigi Ambrosio, Barbara Zavan

**Affiliations:** 1 Maria Cecilia Hospital, GVM Care & Research, Cotignola, 48033 Ravenna, Italy; 2 Institute of Polymers, Composites and Biomaterials—National Research Council (IPCB-CNR), 80125 Naples, Italy; 3 Department of Translational Medicine, University of Ferrara, 44121 Ferrara, Italy

**Keywords:** methacrylated hyaluronic acid, hydroxyapatite, polyetheretherketone, hydrogel, bone substitute

## Abstract

Hybrid bone substitute made up of a 3D printed polyetheretherketone (PEEK) scaffold coated with methacrylated hyaluronic acid (MeHA)-hydroxyapatite (HAp) hydrogel is the objective of the present work. Development and characterization of the scaffold and of the MeHA-HAp after its infiltration and UV photocrosslinking have been followed by analyses of its biological properties using human mesenchymal stem cells (MSCs). Interconnected porous PEEK matrices were produced by fused deposition modeling (FDM) characterized by a reticular pattern with 0°/90° raster orientation and square pores. In parallel, a MeHA-HAp slurry has been synthesized and infiltrated in the PEEK scaffolds. The mechanical properties of the coated and pure PEEK scaffold have been evaluated, showing that the inclusion of MeHA-HAp into the lattice geometry did not significantly change the strength of the PEEK structure with Young’s modulus of 1034.9 ± 126.1 MPa and 1020.0 ± 63.7 MPa for PEEK and PEEK-MeHA-HAp scaffolds, respectively. Human MSCs were seeded on bare and coated scaffolds and cultured for up to 28 days to determine the adhesion, proliferation, migration and osteogenic differentiation. In vitro results showed that the MeHA-HAp coating promotes MSCs adhesion and proliferation and contributes to osteogenic differentiation and extracellular matrix mineralization. This study provides an efficient solution for the development of a scaffold combining the great mechanical performances of PEEK with the bioactive properties of MeHA and HAp, having high potential for translational clinical applications.

## 1. Introduction

Currently, the most widely used materials for orthopedic and dental purposes are metals, primarily titanium and its alloys, due to their valuable intrinsic properties and potential surface modifications [1,2,3,4,5,6]. Metals have numerous advantages, including biocompatibility, corrosion resistance and mechanical strength. However, they also have some disadvantages, such as the radiopacity of titanium alloys in computed tomography (CT) or magnetic resonance imaging (MRI) and the release of potentially harmful metal ions [7]. Due to these limitations, technopolymers have emerged as alternative materials in bone substitute manufacturing in recent years. The term technopolymers identifies all those polymers with high physico-chemical and mechanical characteristics, such as rigidity, toughness, ductility, workability, resistance to high temperatures, static and dynamic loads and aging. In the case of the production of permanent implants, such as bone substitutes, technopolymers can also guarantee the absence of biodegradability [8].

Among the various technopolymers with biomedical use, polyetheretherketone (PEEK) has been proposed for applications in dental, cranio-maxillofacial and orthopedic surgery, especially since, in the 1990s, the US FDA certified PEEK as a suitable material for bone implantation [9]. PEEK is considered an alternative material to titanium for its excellent biocompatibility and radiolucency, which means that it does not create artifacts in CT and MRI images. Consequently, these imaging techniques can be used to monitor healing around PEEK implants without artifacts obscuring nearby tissue images. Furthermore, since PEEK is also chemically resistant, it does not release toxic by-products like metal implants do when they undergo biocorrosion. However, this technopolymer poorly interacts with bone tissue. In fact, PEEK is hydrophobic and does not allow the absorption of proteins to favor cell adhesion. As a result, instead of anchoring themselves to the surrounding bone through a process called osseointegration, PEEK implants tend to be encapsulated by fibrous tissue and/or colonized by bacteria due to the foreign body reaction that occurs after its implant [10,11]. 

To improve the PEEK bioactivity and osteoinduction, several strategies have been proposed, including coating the surface with different materials that can favor the binding with bone. Different types of coatings have been applied to increase the osseointegration of PEEK, including bioactive ions, calcium phosphate, proteins and peptides. Although these modifications have been classified as coatings, they can also affect the surface morphology, chemistry and wettability of PEEK [12].

Among the biomaterials coatings, natural polymers represent an excellent solution for in vitro and in vivo applications [13]. They may include polysaccharides and proteins-based hydrogels, which are polymers with excellent swelling properties and a high affinity for water. Moreover, given the complexity of the bone regeneration process, hydrogel material employed for bone tissue engineering should possess a biomimetic nature table to simulate the ECM architecture [14]. In particular, hyaluronic acid (HA) is a biopolymer with therapeutic applications, which is gaining increased attention as a versatile surface-treatment material for improving device biocompatibility [15]. HA is characterized by a unique set of properties, such as suitable rheological behavior and lubricity. It is involved in many natural processes such as biological signaling, wound repair, morphogenesis and extracellular matrix (ECM) organization. However, its low residence time and fast degradability limit its applicability. For this reason, the ability to be chemicallymodified allows the creation of a wide range of stable bioactive surfaces [16]. Indeed, the photo-crosslinking of HA derivatives, such as methacrylated HA (MeHA), can be considered one of the possible strategies to immobilize HA on a biomedical implant and produce hydrophilic, lubricious, and biocompatible surfaces [17]. Therefore, integrating HA with other more bioactive components, such as Hydroxyapatite (HAp), could increase the mechanical features as well as the bioactivity [14,18]. Indeed, composite hydrogels represent a good strategy for combining the functions of multiple bioactive components. It was demonstrated that hydrogels containing HAp nanoparticles promote higher osteogenic differentiation and mineralization than plain hydrogel [19,20]. Furthermore, recent studies have shown the possibility of realizing nanocomposite materials based on MeHA and calcium phosphates by sol-gel synthesis [18,21]. The overall results highlighted the possibility of tuning the degree of methacrylation of the polymer matrix as well as the inorganic phase concentration of the polymer-based materials, obtaining bio-nanocomposite hydrogels characterized by a wide range of properties for osteochondral tissue regeneration.

Here, we will present a prototype of a hybrid bone substitute made up of PEEK and MeHA-hydroxyapatite (HAp), which can guide cell proliferation offering binding sites, convey nutrients, as well as stimulate the differentiation of mesenchymal stem cells into bone cells. The prototype will combine the excellent mechanical properties of PEEK with the extreme biocompatibility of HA and the osteoinductive and osteoconductive properties of HAp.

## 2. Results

### 2.1. Hyaluronic Acid Methacrylation and Composite Characterization

**Figure 1:**
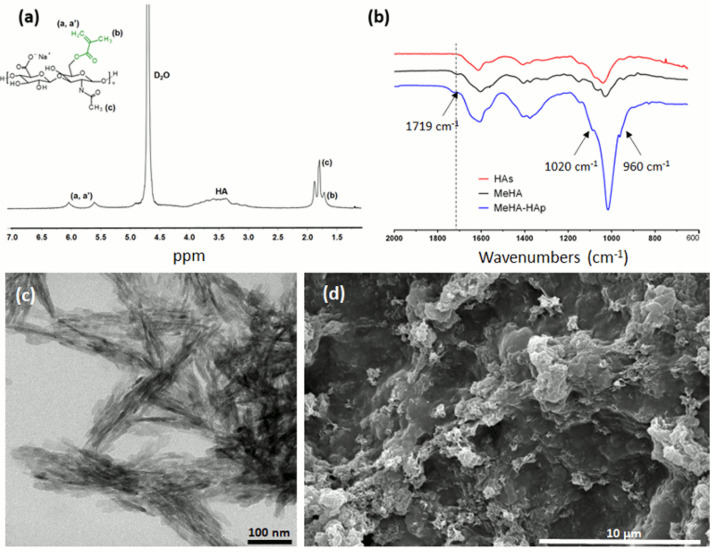
(**a**) Representative ^1^H NMR spectrum of MeHA. (**b**) ATR FT-IR spectra of HAs, MeHA and MeHA-Hap. (**c**) TEM image of HAp. Scale Bar: 100 nm. (**d**) SEM image of MeHA-HAp. Scale Bar: 10 μm.

^1^H NMR spectroscopy confirmed the functionalization of hyaluronic acid with the methacrylate group, showing the relative peaks at 6.04, 5.61 and 1.72 ppm. Figure 1a shows a ^1^H NMR spectrum characteristic for the methacrylation reaction of HA. The degree of substitution (DS) is defined as the amount of methacryloyl groups per disaccharide repeat unit, and it was calculated from the relative integrated intensities of the methacrylate protons (-CH_2_) at 6.04 ppm and methyl protons (-CH_3_) at 1.90. MeHA macromonomers were found to have a DS of approximately 66.1%.

Attenuated total reflection spectroscopy (ATR FT-IR) was performed on neat synthesized polymers and on the composite slurry (Figure 1b). For MeHA, it is possible to observe: the band extending between 950 and 1200 cm^−1^, which corresponds to the C-O stretching vibrations (νC-OH), the intense group of bands from 1500 to 1700 cm^−1^ representing the superposition of amide I and II and of various carbonyl and carboxyl νC=O bands. In particular, the peak at 1719 cm^−1^ belongs to νC=O of the methacrylic moiety, confirming that the functional moieties are successfully branched on the polysaccharide chain. ATR FT-IR analysis was also carried out on the nanocomposite slurry. In the spectrum, the main chemical groups of HAp, PO_4_^3−^, OH^−^, and CO_3_^2−^ are present. In particular, the peak at 1020 cm^−1^ and the shoulder at 960 cm^−1^ are typical of asymmetrical stretching (v3) and bending (v1) modes of PO_4_^3−^. The OH^−^ and CO_3_^2−^ are covered by the typical peaks of MeHA.

From the morphological point of view, SEM and TEM have allowed acquiring information about the size and distribution of HAp inside the polymer matrix (Figure 1c,d). TEM analyses were performed to obtain a magnified image of the gel suspension and quantify the shape and size of the crystals. Figure 1c reports TEM analyses performed on MeHA-HAp.

The crystal showed a length of about 85.9 ± 2.9 nm and a thickness of 4.9 ± 1.8 nm, typical of natural HAp in the bone. SEM analyses were carried out on the surface of the gel suspension. The HAp fillers appeared homogeneously distributed, with no evidence of microscale clusters (Figure 1d).

**Figure 2:**
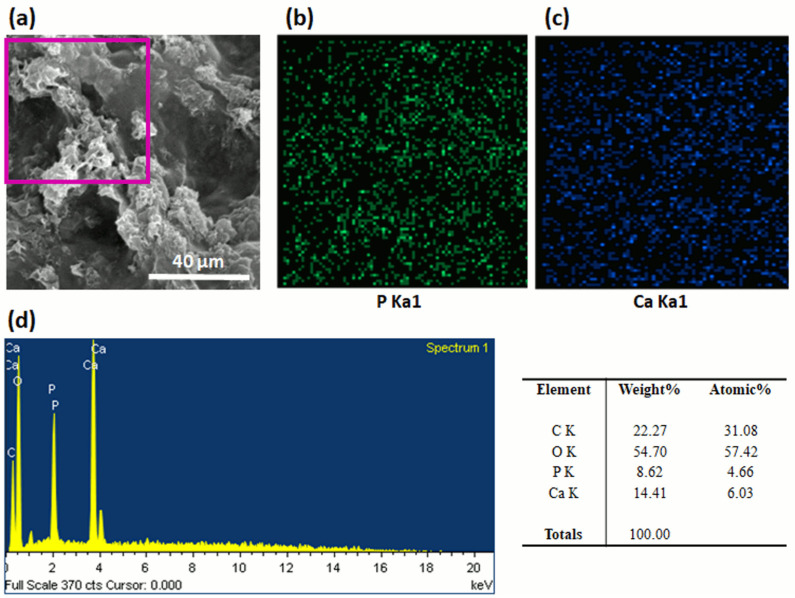
SEM-EDS analysis performed on MeHA-HAp coating: (**a**) SEM with pink box indicating the region of interest; (**b**) EDS mapping of Phosphorous (P); (**c**) EDS mapping of Calcium (Ca) (**d**) elemental analysis.

In order to assess the presence of HAp, an elemental distribution analysis was carried out. SEM–EDS highlighted the presence of Ca and P ions with a molar ratio (Ca/P = 1.67) similar to the typical ratio of natural HAp (Figure 2).

Furthermore, P- and Ca-mapping photographs of MeHA-HAp are presented in Figure 2b,c. Calcium was labeled blue, while phosphorus was labeled green. From the picture, particles appeared homogeneously distributed inside the polymer matrix.

**Figure 3:**
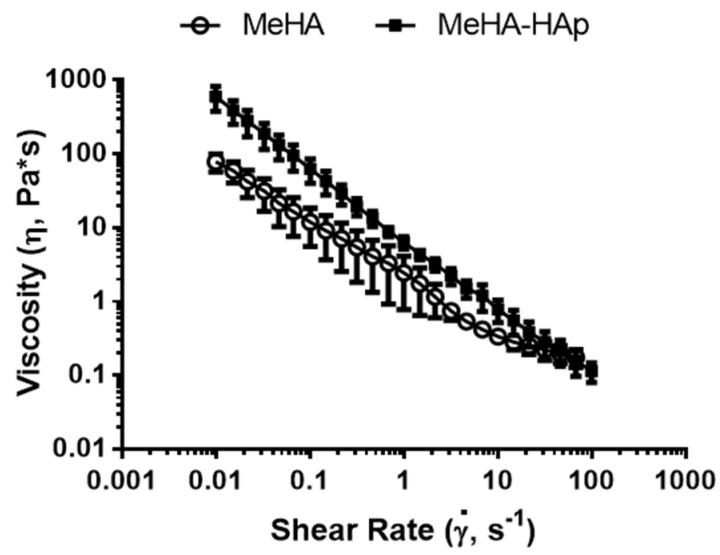
Results from rheological tests on a MeHA and MeHA-HAp: viscosity as a function of shear rate.

The nanocomposite material was also characterized in terms of flow behavior. Characterizing flow behavior across a range of shear rates was imperative to optimize the processing conditions for the PEEK infiltration. One of the most important properties of flow behavior is the measurement of viscosity as a function of shear rate, as this value is directly related to a material’s processability. Results from steady shear tests showed that viscosity decreases as the shear rate increases, highlighting a shear thinning behavior for both formulations (Figure 3). Furthermore, the presence of HAp increased the viscosity of neat MeHA. However, MeHA-HAp was still injectable, as demonstrated by the decrease in viscosity over the shear rate range.

### 2.2. PEEK-MeHA-HAp Bone Substitute: Preparation and Characterization

Interconnected porous PEEK matrices were produced by fused deposition modeling (FDM) printing. To favor the interaction with the bone, the PEEK structures have been molded with controlled porosity. The porous structures better simulate bone morphology and are necessary for the formation of new bone tissue as they improve the adhesion, migration and proliferation of cells, as well as the diffusion of oxygen and substances. Furthermore, the presence of interconnected pores can support neovascularization, which is necessary for the adequate development of bone tissue. Finally, the presence of pores allows the introduction of the MeHA-HAp hydrogel within the technopolymer-based matrix.

**Figure 4:**
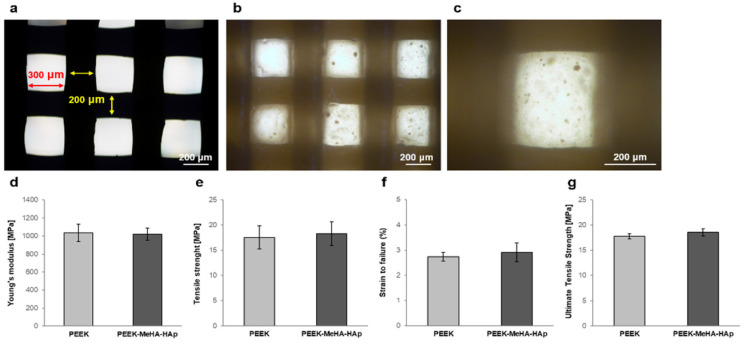
PEEK-MeHA-HAp bone substitute. Phase contrast microscope images of (**a**) bare PEEK matrix with square pores with 300 μm side and 200 μm gap (4× magnification), (**b**) PEEK matrix filled with MeHA-HAp (4× magnification), (**c**) PEEK-MeHA-HAp bone substitute at 10× magnification. (**d**) Young’s modulus, (**e**) tensile strength, (**f**) strain to failure, and (**g**) ultimate tensile strength of PEEK-MeHA-HAp and bare PEEK.

The PEEK matrices were 3D-printed with a reticular pattern where one layer was printed in one direction and the next layer rotated by 90°. Phase contrast microscope images showed the interconnected lattice geometry stably repeated within the entire matrices (Figure 4a). In particular, the PEEK matrix was characterized by square pores with an internal side 300 μm long and a side thickness of 200 μm. In addition, Figure 4b,c shows the successful incorporation of MeHA-HAp within the reticulated PEEK structures. It is interesting to note how the MeHA-HAp nanocomposite was uniformly distributed within the technopolymer mesh. As also clearly expected, the inclusion of MeHA-HAp into the lattice geometry did not significantly change the strength of the PEEK structure. Figure 4d–g reports Young’s modulus, tensile strength, strain to failure, and ultimate tensile strength of PEEK-MeHA-HAp and bare PEEK.

### 2.3. PEEK-MeHA-HAp Bone Substitute: Bioactivity

**Figure 5:**
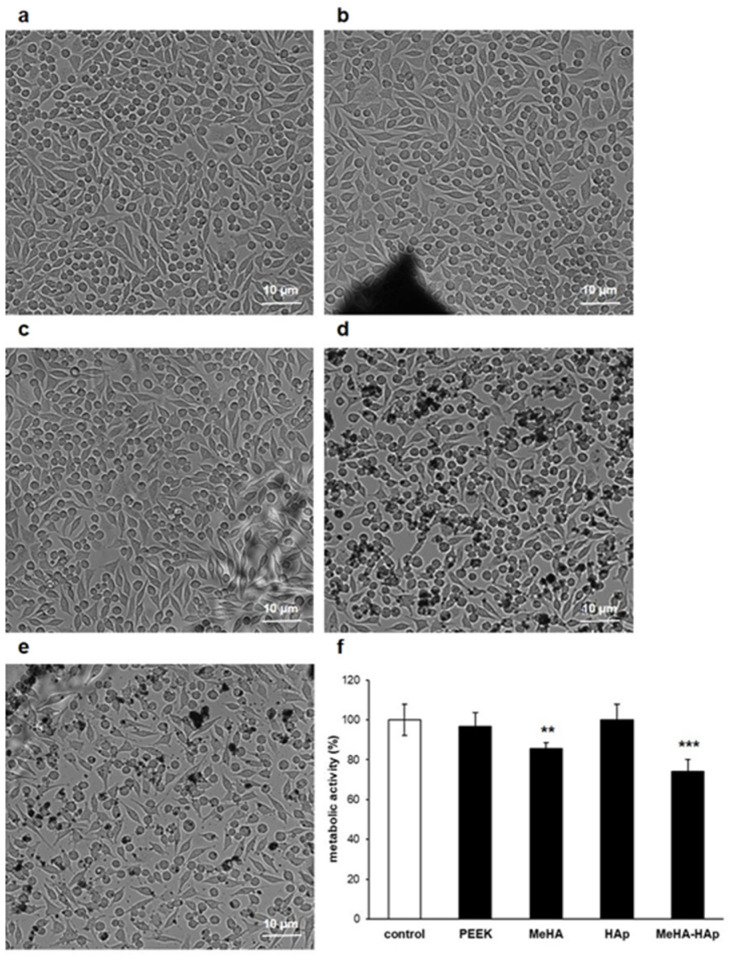
In vitro cytotoxicity test. (**a**–**e**) Representative images of NCTC (murine fibroblasts clone L929) morphology: (**a**) without tested material (control), (**b**) with PEEK, (**c**) MeHA, (**d**) HAp, and (**e**) MeHA-HAp. Scale bar 10 μm. (**f**) Metabolic activity percentage after 24 h. ** *p* < 0.01; *** *p* < 0.001 with respect to the control.

Firstly, the biocompatibility of bone substitute components was evaluated following the ISO 10993-5 standard instruction for assessing the in vitro cytotoxicity [22]. The method involves the direct contact of tested materials with a monolayer of mouse fibroblasts NCTC clone L929. Precisely, once the cell monolayer reached the sub-confluence, the materials were added to cover an area of approximately one-tenth of that occupied by the cellular monolayer. After 24 h of incubation at 37 °C and 5% CO_2_, the interpretation of the results was based both on the qualitative evaluation of cell morphology by microscopic observation and on the quantification of cellular metabolic activity measured with the MTT test. Cells grown in the absence of materials represented the control condition (Figure 5). NCTCs showed no visible signs of distress in any treatment, appearing with the typical round and spindly morphology. Setting the control condition equal to 100.00% (±3.18), the metabolic activity of NCTCs in contact with PEEK was equal to 96.77 ± 2.79%, that in contact with HAp was 100.00 ± 3.18%; for MeHA, it was 85.46 ± 1.24%, while for the MeHA-HAp composite it was 74.00 ± 2.49%. The standard method considers the reduction of cell viability by more than 30% a cytotoxic effect [22]. Therefore, none of the tested materials is cytotoxic, not even MeHA-HAp, which showed the lowest percentage of cell viability (Figure 4e). Although a lower percentage of metabolic activity was recorded, however within the biocompatibility range, the morphology of the cells in contact with MeHA-HAp is comparable to cell morphology in the control condition (Figure 5a,e, respectively), further confirming the non-toxicity of the material.

Subsequently, the bioactivity of the bone substitutes was tested by seeding human MSCs and maintaining the cultures for 28 days in order to determine the adhesion, proliferation, migration and osteogenic differentiation of the mesenchymal cells.

**Figure 6:**
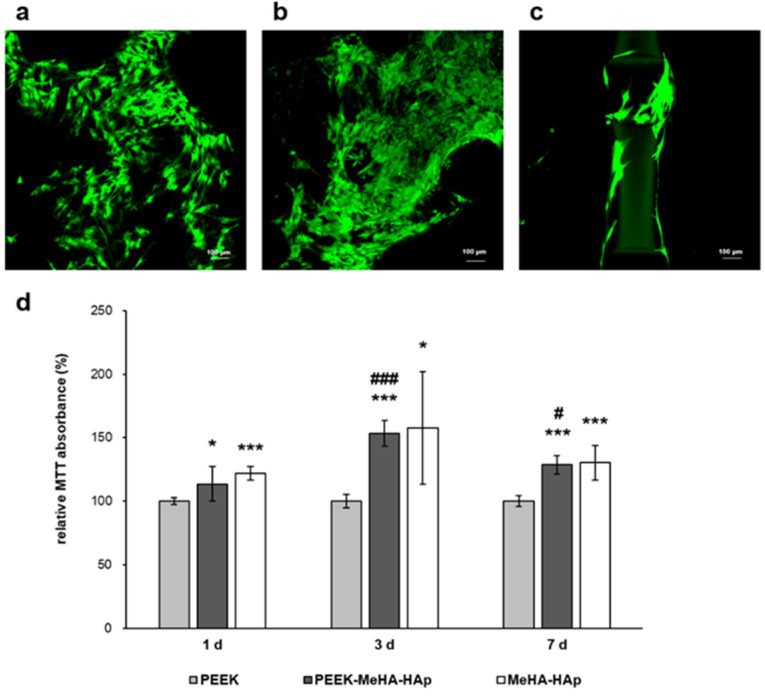
Human MSC adhesion and proliferation. The adhesion was detected by calcein-AM staining one day after the seeding. Confocal microscope images of (**a**) PEEK-MeHA-HAp, (**b**) MeHA-HAp, and (**c**) PEEK. (**d**) Cell proliferation was detected by MTT assay at 1, 3, and 7 days after the seeding. Results are expressed as a percentage of viability referred to MSCs growing on bare PEEK at each time point. * *p* < 0.05; *** *p* < 0.001 with respect to PEEK. # *p* < 0.05; ### *p* < 0.001 with respect to day 1 of culture (1d).

The cell adhesion was monitored by incubation with a calcein-AM probe one day after the seeding. The probe permeates cell membranes in a non-fluorescent form and is converted into green fluorescent calcein by the intracellular esterases. The fluorescence observed under the confocal microscope is, therefore, proportional to cell viability and to the number of attached cells on bone substitutes. Confocal microscope images demonstrated that PEEK-MeHA-HAp bone substitutes ensure greater cell adhesion than bare PEEK and are comparable to the MeHA-HAp hydrogel (Figure 6a–c). Moreover, MSC proliferation was monitored by MTT assay on days 1, 3 and 7 (Figure 6d). A substantial proliferation in each condition occurred throughout the culture period, confirming the absence of toxicity. In general, the absorbances for PEEK-MeHA-HAp and MeHA-HAp were higher than those for bare PEEK at each time point. Compared to the first day of culture (1d), PEEK-MeHA-HAp is the only condition showing a significant increase in proliferation at day 3 (*p* < 0.001) and 7 (*p* < 0.05).

**Figure 7:**
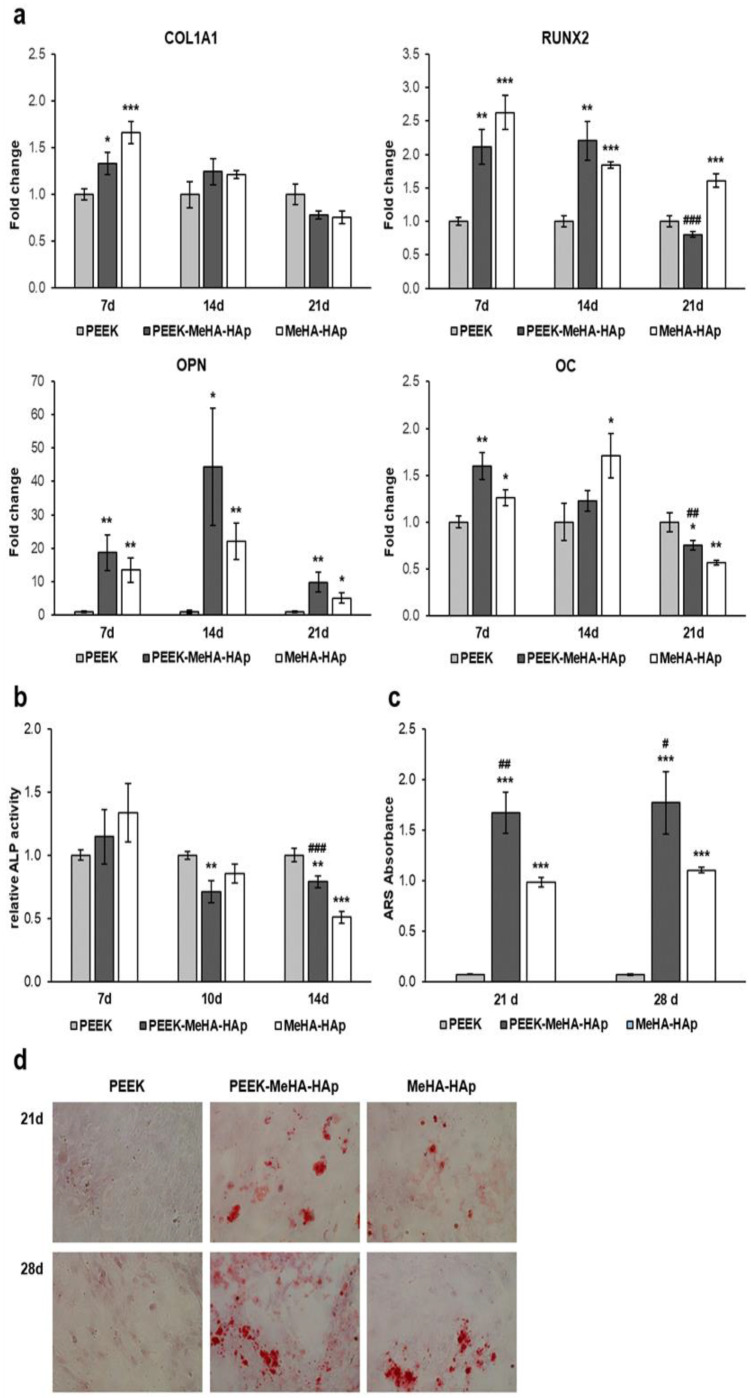
Osteogenic differentiation on PEEK-MeHA-HAp, MeHA-HAp, and PEEK. (**a**) Expression profile of osteogenesis-related genes: COL1A1, RUNX2, OPN, and AC. (**b**) Alkaline phosphatase (ALP) activity. (**c**) Alizarin Red S (ARS) quantification and (**d**) staining. * *p* < 0.05; ** *p* < 0.01; *** *p* < 0.001 with respect to bare PEEK. # *p* < 0.05; ## *p* < 0.01; ### *p* < 0.001 with respect to MeHA-HAp.

The mRNA levels of osteogenesis-related genes, including collagen type I (COL1A1), runt-related transcription factor 2 (RUNX2), osteopontin (OPN), and osteocalcin (OC) were quantified at 7, 14 and 21 days (Figure 7a). On day 7, the expression level of COL1A1, RUNX2, OPN, and OC were significantly higher on PEEK-MeHA-HAp than on bare PEEK. The MeHA-HAp also showed a greater expression of the above genes than PEEK. The same trend was observed after 14 days of culture. At day 21, the expression of OPN was greater, and that of OC was lesser in PEEK-MeHA-HAp compared to PEEK. For MeHA-HAp, the expression of RUNX2 was also greater compared to PEEK. By comparing PEEK-MeHA-HAp and MeHA-HAp, the expression of OPN and OC was greater, and that of RUNX2 was lesser on PEEK-MeHA-HAp. The osteogenic differentiation was also examined through enzymatic and histological assays. The activity of alkaline phosphatase (ALP) was monitored at 7, 10 and 14 days (Figure 7b). Compared to bare PEEK, ALP on PEEK-MeHA-HAp and MeHA-HAp substrates reaches the maximum activity at 7 days. Subsequently, the activity on PEEK-MeHA-HAp maintained higher values than on MeHA-HAp. Moreover, the mineral deposits on each surface were detected and quantified by Alizarin Red S (ARS) staining on days 21 and 28 (Figure 7c,d). The results revealed greater extracellular matrix mineralization levels for PEEK-MeHA-HAp than for PEEK or MeHA-HAp at each time point.

## 3. Discussion

Recently, the technopolymer PEEK has been investigated as an implant material. Although PEEK has several advantageous features, including biocompatibility, chemical stability, and elastic modulus similar to that of natural bone, it is poorly integrated with surrounding bone tissue after implantation. To improve the bioactivity of PEEK, different strategies to functionalize the surface or to change its structure have been proposed. Here, the surface of PEEK was coated with a biocompatible and osteoinductive hyaluronan derivative named MeHA-HAp. As HA is a constituent of the tissue ECM, it plays a constitutive role in cell functions such as adhesion, proliferation, migration, angiogenesis, and wound healing [23,24,25,26]. However, due to the fast degradation rate, HA has found limited applications in tissue engineering. Besides its biological properties, HA also has a useful chemical structure that makes it an attractive biomaterial [27,28,29,30]. The presence of a variety of functional groups, such as carboxyl, hydroxyl, and amide groups, on HA’s backbone, allows for easy modification [31,32]. Chemical modifications may improve the stability of the HA without altering its bioactivity. One of the most used modifications that can improve HA stability is the addition of methacrylate moieties on the HA backbone, which leads to the possibility of creating a covalent network by UV photo-polymerization [18,21,33]. The modification allows for controlled crosslinking, resulting in the fabrication of hydrogels resembling native ECM properties [24,34]. Moreover, MeHA-based nanocomposites can be suitably prepared by the physical embedding of inorganic nanoparticles, such as HAp, in order to also confer osteoinductive properties [35]. In the present work, HA was successfully modified, obtaining a UV photocrosslinkable MeHA, which showed a high degree of substitution (DS~66.1%) and shear thinning behavior. Furthermore, the inclusion of HAp nanoparticles, obtained by sol-gel synthesis, allowed the realization of a nanocomposite injectable hydrogel-based material.

In parallel, PEEK matrices with interconnected porous structures were produced to promote interactions at implant-bone interfaces and to allow a more effective infiltration of MeHA-HAp. Porous implants provide improved biological fixation due to enhanced bone ingrowth into the pores facilitating a higher volume of tissue-implant interaction and efficient load transfer along the interface. Porous structures with high surface area and three-dimensional pore connectivity were clinically proven to promote tissue adhesion, growth and vascularization through the transport of oxygen and nutrients [36]. Moreover, porous structures are necessary for new bone tissue formation as they facilitate the adhesion of MSCs by offering binding sites allowing cell migration and proliferation. Interconnected pores also support the neovascularization necessary for the adequate development of bone tissue [36]. Although porosity degree and pore size in bone implants is crucial for implant stability and integration into the bone tissue, at the same time, they can determine a reduction in the mechanical properties [37]. Therefore, it is essential to find an optimal degree of porosity in order to balance adequate mechanical strength with a positive biological response. It has been reported that pore sizes in the 150–1200 μm range support a positive response from the cells and allow the growth of bone tissue within them. In fact, pores of 75–100 μm determine the growth of non-mineralized osteoid tissue, while even smaller pores (10–44 and 44–75 μm) are penetrated only by fibrous tissue. A minimum size of 300 μm is instead recommended to allow vascularization and cell hosting [38,39,40,41]. Based on these considerations, the present PEEK matrix was fabricated with a reticular pattern in which square interconnected pores have an internal side long 300 μm and a side thickness of 200 μm. Microscopy evaluation demonstrated the effective deposition of MeHA-HAp coating on the surface and inside the pores of the scaffold. Moreover, MeHA-HAp does not change the elastic modulus of the PEEK matrix, as demonstrated by mechanical tests. Conversely, cell adhesion and proliferation are enhanced on PEEK-MeHA-HAp compared to the bare PEEK surface. The improved cell adhesion and proliferation may depend on enhanced surface hydrophilicity resulting from the hyaluronan-derivative coating. Indeed, it is demonstrated that hydrophilic surfaces are more conducive to cell adhesion and proliferation compared to hydrophobic surfaces [42]. Additionally, coatings made up of hyaluronan derivatives determine the adsorption of extracellular protein, which further favors cell adhesion and then the interaction with the scaffolds [43]. The quick interaction between host cells and implant surface is pivotal for prompt bone formation and, therefore, for implant stability. The HAp inside the coating has a dual objective, to favor cell adhesion as well as osteogenic differentiation. In fact, HAp nucleation sites can precipitate apatite crystals to promote cell attachment and growth and osteo-differentiation of MSCs, inducing the progression of a new mineral matrix [44,45,46]. The proliferation profile of human MSCs recorded on MeHA-HAp and PEEK-MeHA-HAp agrees with the dual role of hydroxyapatite nanoparticles. In fact, contrary to bare PEEK, these substrates determine a greater proliferation of cells from the first days. Subsequently, the induction to osteogenic differentiation determined a mild reduction in cell proliferation [47]. The osteo-differentiation of MSCs on the PEEK-MeHA-HAp surface is crucial for bone regeneration and the success of the bone substitute prototype. Therefore, osteogenesis-related gene expressions, ALP activity, and ECM mineralization are evaluated in vitro. Precisely, the expression of COL1A1, RUNX2, OPN, and OC were examined by RT-PCR. RUNX2 is one of the most specific genes at the early stage in terms of osteoblastic differentiation, and it triggers COL1A1 and OC expression, which are important bone matrix proteins [48]. COL1A1 is an early marker of osteoblast commitment, and it is one of the major proteins in the ECM. The high expression of COL1A1 represents an active osteoblastic differentiation. OC is a late-stage marker of osteogenic differentiation, whereas OPN is one of the phosphoproteins which plays a crucial role in the mineralization and absorption of the bone matrix [49]. ALP is an enzyme necessary to bone formation as it regulates inorganic phosphate metabolism. ALP hydrolyzes phosphate esters and increases the local concentration of phosphates, thus promoting the mineralization of ECM. Therefore, it is considered a marker of early osteogenic differentiation. When cells enter into the mineralization step, mineralized ECM begins to deposit, and its buildup is considered the marker for late osteogenic differentiation [50]. Overall, MSCs on PEEK-MeHA-HAp exhibited osteogenic differentiation at the genetic and molecular levels. In fact, on the seventh day, the first stage of osteogenic differentiation in our experimental setting, PEEK-MeHA-HAp determines a greater activity of ALP enzyme and a greater expression of the markers RUNX2, COL1A1, OC, and OPN compared to bare PEEK. On the fourteenth day, the expression of the osteogenesis-related genes remains higher than those on bare PEEK. On the other hand, during the late phase of osteogenic differentiation, both a reduced expression of early markers and a higher mineralized matrix were observed. In particular, high levels of expression for OC and OPN and low for RUNX2 were observed on PEEK-MeHA-HAp rather than on MeHA-HAp coating. Since the expression of the transcription factor RUNX2 is primary for the activation of other osteogenesis-related genes, such as OC and OPN, while undergoing a reduction in the late phase of differentiation, it can be hypothesized that osteogenic differentiation on PEEK-MeHA-HAp occurs in advance. This also correlates with the greater deposition of the mineralized matrix recorded at 21 and 28 days. Collectively, the gene expression, histological and enzymatic results showed that the PEEK-MeHA-HAp scaffold significantly promoted [18,21,33] osteogenesis when compared with neat PEEK or MeHA-HAp coating.

## 4. Materials and Methods

### 4.1. Synthesis and Characterization of MeHA-HAp

MeHA. Hyaluronic acid sodium salt (HAs, M_w_ = ~340 kDa from *Streptococcus equi*, Bloomage Freda Biopharm Co. Ltd., Shandong, China) was modified to graft photoactive polymerizable groups by reacting with methacrylic anhydride (Me, Sigma Aldrich Milano , Italy). MeHA was synthesized following an adapted protocol [18,21,33,51]. Briefly, 1 g of HAs was dissolved in 10 mL of distilled water (H_2_O) and stirred at room temperature for complete dissolution. Methacrylation was obtained by reacting the primary hydroxyl groups (-OH) with Me at 4 °C, keeping the pH between 8 and 9 using a sodium hydroxide solution (NaOH, Sigma Aldrich). An excess of 30 mol% Me per (-OH) has been used to reach a degree of substitution (DS) of 85%. The reaction was carried out overnight. When the reaction was completed, MeHA solutions were precipitated into cold anhydrous ethyl alcohol (EtOH), and the supernatant was recovered by vacuum filtration. The isolated MeHA polymers were then dialyzed against pure water for 5 days and freeze-dried (CoolSafe™ 55-4 PRO).

**Scheme 1:**
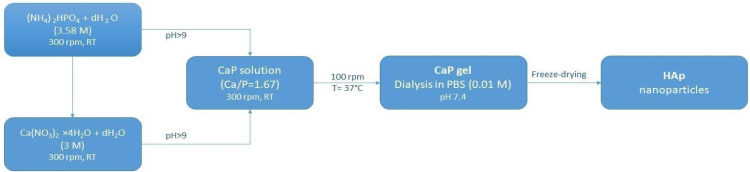
Preparation of HAp at room temperature by sol-gel approach.

HAp. The synthesis of HAp was based on the sol-gel approach through hydrolysis and condensation reactions. For that, calcium nitrate tetrahydrate (Ca(NO_3_)_2_ × 4H_2_O, Sigma Aldrich, Milano, Italy) and diammonium hydrogen phosphate ((NH_4_)_2_HPO_4_, Sigma Aldrich, Italy) were used as precursors of Ca^2+^ and PO_4_^3-^ respectively, whereas H_2_O as solvent. The synthesis took place at room temperature in an alkaline environment (pH 9–11) by adding ammonium hydroxide solution (NH_4_OH, Sigma Aldrich). The reaction steps are summarized in Scheme 1. Finally, to obtain the MeHA-HAp nanocomposite slurry, MeHA (20 mg/mL) was dissolved in H_2_O containing Irgacure 2959 (Sigma Aldrich) at a concentration of 0.1 % (*w/v*). HAp nanoparticles were added to the MeHA solution (10 mg/mL) under vigorous stirring for 30 min to obtain a homogeneous dispersion.

^1^H Nucelar magnetic resonance. ^1^H Nuclear magnetic resonance (NMR, Bruker AVIII 400HD, Fällanden, Switzerland) was employed to calculate the DS of the MeHA. MeHA (6 mg/mL) was completely dissolved in deuterium oxide (D_2_O) by using a vortex mixer, and it was transferred into NMR tubes. The data were collected at a frequency of 400 MHz. Phase and baseline corrections were applied before obtaining the areas (integrals) of purely absorptive peaks. The DS of MeHA was determined by comparing the integral area of protons of unsaturated C=C bonds (methacrylated moieties) with methyl (–CH_3_) peak.

Attenuated total reflection spectroscopy. ATR FT-IR (ThermoFisher Nicolet IS10 with a Zinc selenide (ZnSe) crystal material) was used to identify the functional groups introduced on the backbone of HAs and the presence of HAp into the polymer matrix.

Dried MeHA and MeHA-HAp (6 mg) were scanned from 600 to 2000 cm^−1^ with a resolution of 2 cm^−1^. Furthermore, the neat HAs powder was scanned under the same conditions.

TEM. Images were collected using a Hitachi H-9000NAR model instrument operated at an accelerating voltage of 100 kV. Samples were prepared by placing a drop of the composite gel suspensions (diluted in H_2_O and dispersed by ultrasonic waves) onto carbon-coated copper grids, dried in air and loaded into the electron microscope chamber.

SEM. The composite injectable hydrogel was observed by SEM (S-800; Hitachi, Tokyo, Japan). Before the analysis, MeHA-HAp was prepared as described before, frozen, lyophilized (CoolSafe™ 55-4 PRO) for 48 h. The lyophilized material was coated with an ultrathin layer of Au/Pt by using an ion sputter and then observed by SEM. SEM-energy dispersive X-ray spectroscopy (EDS) analysis and mapping were also used to assess the chemical composition and dispersion of HAp in the polymer matrix.

Steady shear measurements. The viscosity as a function of the shear rate was evaluated through steady-state shear measurements performed on the MeHA and MeHA-HAp hydrogel solutions. All the measurements were carried out at room temperature in a wide range of shear rates (0.01–100 s1), using a rheometer (HAAKE MARS Rheometer) with a parallel plate/cone 1/60° geometry.

### 4.2. Production and Characterization of PEEK-MeHA-HAp

PEEK matrices were manufactured by an FDM printing device (G3 XP, Gimax 3D, Prato, Italy) featuring a print volume of 400 × 400 × 400 mm and a heatable print bed up to 140 °C. The printing process was defined by the Simplify3D software package. The parameters of the printing process include a nozzle with a diameter of 0.4 mm, a nozzle temperature of 410 °C, and a printing bed temperature of 100 °C. The speed of movement of the nozzle and the temperature of the nozzle has been set to favor the adhesion of the porous structures under construction to the printing surface. After fabrication, the chamber was cooled to room temperature by natural convection, while the molded structures were removed from the build plate by hand. Finally, a MeHA-HAp solution (20 and 10 mg/mL, respectively) was easily infiltrated into the pores of the structures. Afterward, the structure PEEK-MeHA-HAp was photocrosslinked by UV light radiation for 5–10 min.

The tensile tests were performed following the ISO 527-1: 2012, ISO 527-2: 2012: Plastics-Determination of tensile properties concerning the polymeric plastic materials. The test was performed with 7 type 1BA specimens using the MTS Acumen 3 Electrodynamic Test Systems instrument with a 3kN load cell (MTS Systems Corporation, Eden Prairie, MN, USA). The specimens were kept in a “standard atmosphere” for at least 16 h before the test.

The in vitro cytotoxic potential of materials was evaluated through direct contact with murine fibroblasts (NCTC clone L929 mouse, ATCC, Manassas, VA, USA). The test was performed following the ISO 10993-5 standards instructions, as published elsewhere [47]. Briefly, subconfluent monolayer murine fibroblasts were incubated at 37 °C for 24 h with test materials. Cell cultured without tested material represented the control. The cytotoxicity of materials was then examined both microscopically and by quantifying the cell metabolic activity using MTT (Sigma-Aldrich) assay.

### 4.3. Cell Seeding, Adhesion and Proliferation Assay

Human MSCs were seeded with a density of 2 × 10^5^ cells per piece in the osteogenic differentiative medium. The medium consists of Dulbecco’s Modified Eagle Medium (DMEM, Euroclone, Milano, Italy), supplemented with 1%Penicillin/Streptomycin (P/S, EuroClone), 10 % Fetal Bovine Serum (FBS, Life Technologies, San Mateo, CA, USA), 10 mM β-glycerophosphate (Sigma-Aldrich, MO, USA), 100 nM dexamethasone (Sigma-Aldrich), and 200μM ascorbic acid (Sigma-Aldrich). The cultures were maintained for up to 28 days at 37 °C and 5% CO_2_ changing medium every two days.

On the day following seeding, the cell adhesion was detected by staining with 1 μM Calcein AM (Life Technologies, Chagrin Falls, OH, USA) for 20 min at 37 °C and 5% CO_2_. After removing the excess probe, a laser scanning confocal microscopy system (Nikon A1 confocal microscope, Nikon Corporation, Tokyo, Japan) equipped with a 10× objective was used for image acquisition.

Cell proliferation was detected after 1, 3 and 7 days from seeding with MTT (methylthiazolyl-tetrazolium)-based proliferation assay. Samples were incubated with 0.5 mg/mL MTT solution for 3 h at 37 °C and 5% CO_2_. After removing the MTT solution, formazan salt was extracted from each sample with 10% DMSO in isopropanol for 30 min at 37 °C. Absorbance values at 570 nm were recorded in duplicate on aliquots deposited in a 96-well plate using the multilabel plate reader Multiskan FC (Thermo Fisher Scientific, Waltham, MA, USA) [52]. Cell proliferation was represented as the proliferation percentage normalized to the value measured for bare PEEK, which was assigned a value of 100%.

### 4.4. Real-Time PCR

Total RNA was isolated with the RNeasy Mini Kit (Qiagen, Hilden, Germany). The RNA quality and concentration of the samples were measured with the NanoDrop™ 2000 (Thermo Fisher Scientific). 100 ng of total RNA was reverse-transcribed using the QuantiNova™ Reverse Transcription Kit (Qiagen) using SimpliAmp™ Thermal Cycler (Thermo Fisher Scientific). Real-time PCR was carried out using 700 nM primers. And QuantiNova SYBR Green PCR (Qiagen) on a StepOnePlus™ Real-Time PCR System (Thermo Fisher Scientific). Primers were selected with the tool Primer-BLAST. The following primer sequences were used: COL1A1 (FW: 5′-TGAGCCAGCAGATCGAGA-3′; REV: 5′-ACCAGTCTCCATGTTGCAGA-3′); GAPDH (FW: 5′-GCCGTCTAGAAAAACCTGCC-3′; REV: 5′-AAAGTGGTCGTTGAGGGCAA-3′); OC (FW: 5’-GCAGCGAGGTAGTGAAGAGAC-3′; REV: 5′-AGCAGAGCGACACCCTA-3′); OPN (FW: 5′-TGGAAAGCGAGGAGTTGAATGG-3′: REV: 5′-GCTCATTGCTCTCATCATTGGC-3′); RUNX2 (FW: 5′-AGCCTTACCAAACAACACAACAG-3′; REV: 5′-CCATATGTCCTCTCAGCTCAGC-3′). Data analysis was performed by the 2ΔΔCt method and using glyceraldehyde 3-phosphate dehydrogenase (GAPDH) as an internal reference. Data are presented as mean fold change with respect to the control condition (bare PEEK).

### 4.5. ARS Staining and Quantification

Samples were fixed with 10% formalin (Sigma-Aldrich) for 10 min at room temperature (RT) and washed with water. The staining was performed by incubating samples with 40 mM ARS solution (Millipore, Burlington, MA, USA) for 20 min at RT with shaking. After removing the ARS solution, samples were washed with water 4 times and with PBS for 15 min. The stain was extracted by incubating 0.5 mL of 10% acetic acid (Sigma-Aldrich) for 20 min at RT with gentle agitation. Aliquots of 200 uL were transferred in a 96-wells plate and measured at 405 nm in the plate reader Multiskan FC [53].

### 4.6. ALP Activity

Intracellular alkaline phosphatase (ALP) activity was detected using a commercial kit (Abcam, Cambridge, UK) that uses the phosphatase substrate p-nitrophenyl phosphate (pNPP), which turns in yellow when dephosphorylated by ALP [54]. Briefly, samples were washed, homogenized, and centrifuged at 15,000× *g* for 15 min at 4 °C to remove debris. Lysates were incubated with pNPP for 60 min at RT, and absorbance was measured at 405 nm using the plate reader Multiskan FC. ALP activity of the test samples was calculated as follows:

ALP activity (U/mL) = (B/Δ*T*∗*V*)∗*D* (1)

where B is the amount of pNP in the sample well calculated from the standard curve (μmol), Δ*T* is the reaction time (60 min), *V* is the amount of sample added into the reaction well (in mL), and *D* is the sample dilution factor.

### 4.7. Statistical Analysis

For mechanical characterization, five specimens were tested in triplicate. For biodegradability, biocompatibility, cell migration and proliferation, and gene expression, three specimens were tested in duplicate. Results are presented as mean ± standard deviation (SD) of independent measurements. Statistical analysis of variance of the means was assessed by two-way ANOVA followed by the Bonferroni test with multiple comparisons between the different groups by using GraphPad Prism software (2365 Northside Dr. Suite 560 San Diego, CA 92108—version 7.0).

## 5. Conclusions

Over the past few years, PEEK has been gaining increased attention as a technopolymer and possible candidate material replacing titanium due to its biocompatibility, the physico-chemical properties (i.e., radiolucency and chemical resistance). However, its hydrophobicity negatively affects cell adhesion and differentiation, causing the formation of fibrous tissue, which can also be colonized by bacteria. The novelty of the present work mainly relies on the use of a bioactive coating based on a methacrylate hyaluronic acid-hydroxyapatite (MeHA-HAp) injectable biomaterial. In particular, it was infiltrated into the pores of a 3D printed PEEK structure and UV photocrosslinked to improve the stability and extend its residence time. This work showed quite high importance from a bioengineering point of view with the development of a more reliable, robust and physiologically relevant bone substitute. In particular, the nanocomposite structure of the bone was mimicked by a hybrid combination of different materials: PEEK, MeHA and HAp. PEEK acted as mechanically competent support material, MeHA as a component of the extracellular matrix regulating tissue responses and HAp as the major mineral component of bone. Despite the limitations of the present research, the following conclusions were reached:

- The presence of a MeHA-HAp coating did not influence the mechanical properties of the neat scaffold. Conversely, it synergically improved the hydrophilicity of the PEEK surface and the biological behavior thanks to the polymer MeHA and the bioactive molecule HAp, respectively.
- MeHA-HAp coated PEEK scaffold supports a significant increase in cell adhesion and proliferation than bare PEEK.
- The MeHA-HAp coating on PEEK promotes osteogenic differentiation thanks to the increased expression of osteogenesis-related genes and the increased ALP activity after 7 days, promoting greater mineralization of the extracellular matrix already at 21 days.

For future perspectives, the possibility of introducing further functionalities (i.e., pro-angiogenetic factors and/or antibacterial molecules) will be considered, the aim being to develop the next generation of PEEK bone scaffolds with smart hydrogel coatings.

## Data Availability

Not applicable.
